# Synthesis and Functions of Jasmonates in Maize

**DOI:** 10.3390/plants5040041

**Published:** 2016-11-29

**Authors:** Eli J. Borrego, Michael V. Kolomiets

**Affiliations:** Department of Plant Pathology and Microbiology, Texas A&M University, College Station, TX 77843, USA; eli.borrego@tamu.edu

**Keywords:** jasmonic acid, maize, lipoxygenase, oxylipins, plant-insect interactions, plant-microbe interactions

## Abstract

Of the over 600 oxylipins present in all plants, the phytohormone jasmonic acid (JA) remains the best understood in terms of its biosynthesis, function and signaling. Much like their eicosanoid analogues in mammalian system, evidence is growing for the role of the other oxylipins in diverse physiological processes. JA serves as the model plant oxylipin species and regulates defense and development. For several decades, the biology of JA has been characterized in a few dicot species, yet the function of JA in monocots has only recently begun to be elucidated. In this work, the synthesis and function of JA in maize is presented from the perspective of oxylipin biology. The maize genes responsible for catalyzing the reactions in the JA biosynthesis are clarified and described. Recent studies into the function of JA in maize defense against insect herbivory, pathogens and its role in growth and development are highlighted. Additionally, a list of JA-responsive genes is presented for use as biological markers for improving future investigations into JA signaling in maize.

## 1. Importance of Maize as a Crop and a Genetic Model

Despite contributing over 50% of the annual calories for humans [1] and 34% of the production for animal feed [2], little is known about fundamental hormone biology in monocot plants compared to greater advances with dicot plants, primarily *Arabidopsis*. Maize is a diploid large grain cereal possessing unique reproductive organs where the male and female sexual organs are spatially separated. It also has a rich cultural heritage. Of all of the top most cultivated monocot crops, maize (*Zea mays* L.) is the only one with New World origins. Several characteristics of maize facilitate its use as a model organism to explore fundamental processes: the maize genome was recently sequenced for the B73 [3] and Palomero Toluqueño [4] lines; its separated sexual organs allow for effortless controlled pollination; it possesses tremendous genetic diversity; and it is backed by an intensely collaborative scientific community [5]. 

Maize and its closest crop relative, *Sorghum bicolor*, belong to the Panicoideae subfamily of the grasses [6] and utilize C4 carbon metabolism, an adaptation to high light intensities, temperatures and low water availability [7]. In contrast, most other economically-important cereals, such as wheat, rice and barley, belong to the Pooideae subfamily and utilize the more common C3 carbon metabolism. In recent years, scientific communities have sought to establish *Brachypodium distachyon* [8] and *Setaria viridis* [9] as monocot models for the Pooideae and Panicoideae, respectively [10]. Modern maize arose from an ancient hybridization event between two closely related diploid species that resulted in doubling of the genome (i.e., allopolyploidization) between 8.5 and 13 million years ago [11]. Eventually, the genome of this ancestor was reduced to the diploid state, but with uneven gene loss [3]. This resulted in many maize genes, including jasmonic acid (JA) biosynthesis genes, occurring as pairs throughout the genome. 

## 2. Jasmonates Belong to Oxylipins, a Group of Signals Better Understood in Mammals

JAs belong to the immense group of oxygenated fatty acid products known collectively as oxylipins. Oxylipins are derived from either enzymatic or autoxidation of free or membrane-esterified fatty acids. Since all metabolic processes in cells are enclosed within fatty acid-containing membranes, oxylipins are universally produced across all kingdoms of life. Undeniably, oxylipins are best understood in mammals and in these organisms are termed eicosanoids. Eicosanoids are further categorized into subgroups based on their chemical structure and the enzymes (e.g., lipoxygenases, cyclooxygenases and epoxygenases) that catalyze the fatty acid oxygenation. The major subgroup of enzymatically-derived mammalian oxylipins are leukotrienes, prostaglandins, prostacyclins, thromboxanes, lipoxins, eoxins, hydroxyeicosatetraenoic acids (HETEs) and epoxyeicosatrienoic acids (EETs), while chemically-produced eicosanoids are known as isoprostanes [12]. In mammals, oxylipins regulate diverse physiological processes, such as vasoconstriction, vasodilation, pain response and fever generation. A central characteristic of eicosanoid activity is their cell-type-dependent responses, which occur from the receptor-ligand binding of specific eicosanoid with its particular receptor [13]. In addition to receptor-ligand signaling, oxylipins also possess direct antimicrobial activity [14] and are capable of altering cellular redox status [15].

**Figure 1:**
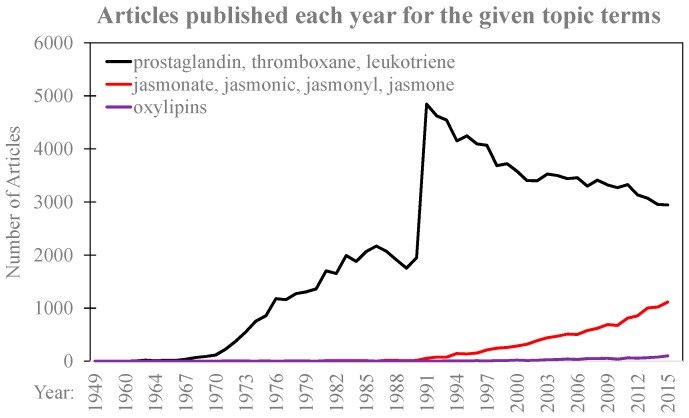
Articles published each year for the given topic terms related to mammalian and plant oxylipins. The x-axis represents year from 1949 to 2015 and y-axis represents the number of articles published each year for the selected topic terms. The line representing the terms related to mammalian oxylipins (terms “prostaglandin”, “thromboxane”, and “leukotriene”), known as eicosanoids, and is colored black. The line representing jasmonates (terms “jasmonate”, “jasmonic”, “jasmonyl”, and “jasmone”) is colored red. Plant oxylipins (term “oxylipins”) is colored purple. Terms were searched in the Web of Science^TM^ citation indexing service and organized per year.

Eicosanoids were first identified in 1935 with the discovery of prostaglandins in seminal fluid [16,17]; however, significant strides in eicosanoid function did not occur until after the development of synthetic eicosanoid standards and improved analytical techniques. Figure 1 represents the number of articles published each year from 1949 through 2015 as indexed by ISI Web of Science using eicosanoids (terms used: “prostaglandin”, “thromboxane” and “leukotrienes”), jasmonates (terms used: “jasmonate”, “jasmonic”, “jasmonyl” and “jasmone” [18]) and plant oxylipins (term used: “oxylipins”) and related oxylipin terms. The first dramatic increase in the number of articles published with the terms associated with eicosanoids in the early 1970s corresponds to the development of methods of the synthesis of prostaglandins [19]. Another substantial increase in the early 1990s coincides with the development of electrospray ionization, an essential component for the detection of biomolecules through mass spectrometry [20]. 

Together, these techniques have allowed for the elucidation of the mechanisms behind the activity of oxylipins on numerous human physiological processes, which has resulted in the understanding of oxylipins and their biosynthetic enzymes as important therapeutic targets in modern clinical medicine. The pharmacological manipulation of oxylipin biosynthesis has been used to relieve fever and pain for several hundred years [21]. The first of these therapies arose from plant extract enriched in salicylic acid and later with synthetic salicylates (e.g., aspirin) and nonsteroidal anti-inflammatory drugs (NSAIDs; e.g., ibuprofen), all of which inhibit cyclooxygenase activity [22]. Selective inhibitors of oxylipin biosynthetic enzymes [23] or substrate availability (e.g., glucocorticoids) [24] have been developed and are routinely prescribed or used over-the-counter. Cyclooxygenase inhibitors make up one third of the prescriptions and 60% of drug costs [25]. Impressively, nearly 80% of the U.S. Food and Drug Administration-approved drugs [26] target pathways under oxylipin regulation.

## 3. Plant Oxylipins

In plants, oxylipins are generated primarily from the enzymatic oxygenation of polyunsaturated fatty acids, linoleic (C18:2) or linolenic acid (C18:3) via lipoxygenase (LOX) or alpha-dioxygenase [27,28]. Chemical oxygenation of fatty acids produces a subset of oxylipins, known as phytoprostanes and phytofurans [29,30]. The structural similarities of mammalian prostaglandins and JA provided plant biologists a springboard to explore the role of oxylipins in the plants and to explore the commonality of oxylipin biology in both kingdoms. Of the more than 600 estimated oxylipins [31] produced from plants as a whole, JA has been in the limelight for nearly all investigations into plant oxylipins. Despite this focus, in sharp contrast to human oxylipins, the number of articles published about JA lags behind (Figure 1). Nonetheless, JA remains the best-understood plant-derived oxylipin and serves as the model oxylipin species in plant biology. Its biosynthesis, signaling and roles in defense, development, tolerance, growth and symbiotic interactions were identified through work with *Arabidopsis*, tobacco and tomato [32,33].

It is important to point out that JA biosynthesis has been reported to occur in fungi from several genera, including pathogenic and symbiotic species [34,35,36]. It appears that at least for some fungi, JA biosynthesis evolved independently, but developed in a manner similar to plant JA production [37]. The capability of fungi to produce JA suggests that fungi modify plants into more suitable hosts through JA-mediated signaling. This phenomenon is similar to how coronatine, a JA structural mimic, is utilized by pathogenic bacteria to facilitate pathogenesis. In that system, coronatine facilitates stomata opening for bacterial entry into the plant [38] and suppresses plant inducible defenses [39]. In turn, JA produced by fungi may be used as molecular cues for defense against diverse pathogens [40]. 

## 4. Jasmonate Biosynthesis in Maize

**Figure 2:**
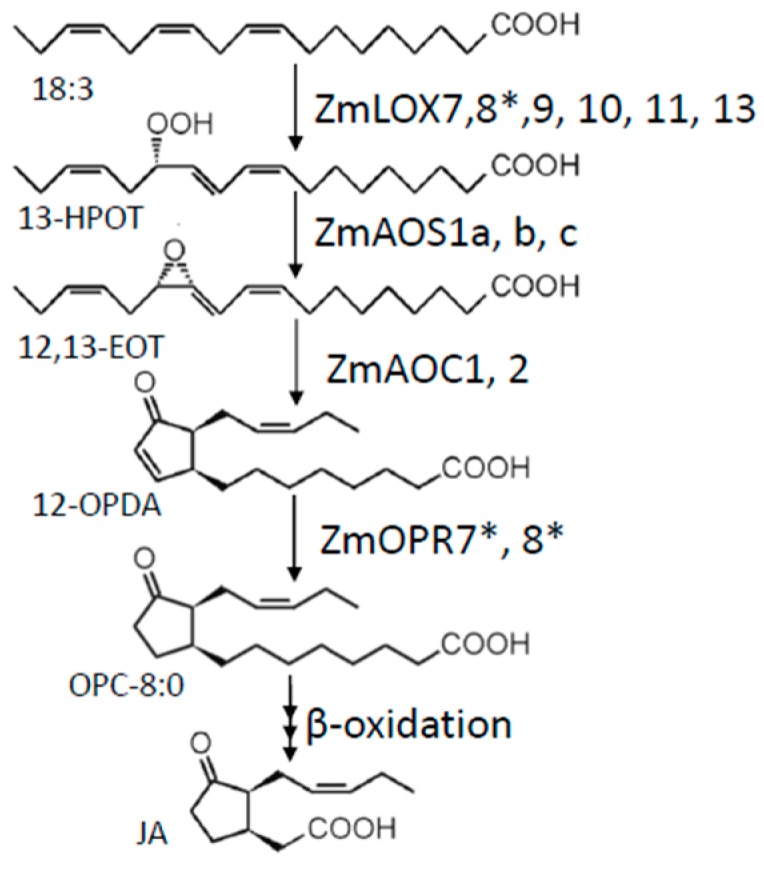
Overview of maize JA biosynthetic pathway. Genes marked with * have been functionally characterized [44,45] and other genes are predicted from phylogenic analysis.

Less than a handful of JA biosynthesis enzymes have been characterized in maize thus far. This review describes the current literature regarding the genes identified via genetic or recombinant protein evidence, but also proposes additional uncharacterized gene candidates with a strong likelihood of their involvement in JA biosynthesis. The majority of studies in JA production arose from investigations utilizing the B73 yellow dent inbred line [41], the first maize genome that has been sequenced. As such, this section regarding JA biosynthesis is B73-centric, and it is reasonable to expect that diverse inbred lines and landraces possess varying copy numbers for specific JA biosynthesis gene family members. Another gene in the octadecanoid pathway, *LIPOXYGENASE5* (*ZmLOX5*), was shown to be present in zero, one and two copy numbers in the inbred lines tested [42]. A diagram of JA biosynthesis with maize-specific enzymes is presented (Figure 2), but for a more detailed description of JA biosynthesis in plants, please refer to the comprehensive historical and current reviews ([32,33,43]).

## 5. 13-Lipoxygenases

**Figure 3:**
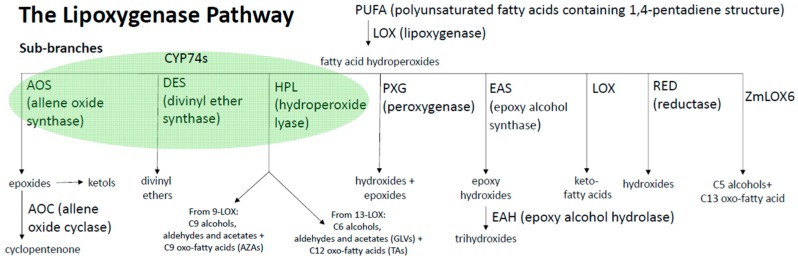
Major metabolite classes produced by the eight sub-branches in the Lipoxygenase (LOX) pathway. LOXs catalyze the dioxygenation of polyunsaturated fatty acids (PUFA), namely linoleic (C18:2) or linolenic (C18:3) fatty acids at the 9- or 13-carbon position. The subsequent hydroperoxide fatty acid can be fluxed into seven sub-branches [31,46]. Allene oxide synthase (AOS), divinyl ether synthase (DES), and hydroperoxide lyase (HPL) are cytochrome P450 monooxygenases (CYP) and part of the CYP74 clade (green oval). The AOS sub-branch is responsible for production of jasmonates (JAs). Divinyl ether synthases (DES) have been identified in some dicots, but not in monocots, including maize. The hydroperoxide lyase (HPL) sub-branch mediates the cleavage of 13-hydroperoxide octadecadi(tri)enoic (HPOD/T) into C6- alcohols, aldehydes, and acetates collectively known as green leaf volatiles (GLVs), and as well as the oxo-fatty acid, traumatin which oxidizes into the dicarboxylic acid, traumatic acid (TA). 9-HPL converts 9-HPOD/T to produce C-9 alcohols, aldehydes, acetates, and C9-oxo-fatty acid that can be oxidized into the dicarboxylic acid, azelaic acid (AZA). Peroxygenase (PXG) catalyzes the formation of hydroxides and epoxides. LOX activity on hydroperoxides yields keto fatty acids. Epoxide alcohol synthase (EAS) synthesizes epoxy-hydroxides which are hydrolyzed to trihydroxy fatty acids. Hydroxides can be converted nonenzymatically from hydroperoxy fatty acids or through action of reductases. ZmLOX6 is a unique monocot specific sub-branch which produces a C13-oxo-fatty acid and C5 alcohols from 13-HPOT specifically [48].

These non-heme iron containing oxygenases are the namesake for an immense pathway of fatty acid metabolism, known as the lipoxygenase (LOX) pathway, consisting of at least seven enzyme branches [46]. Depending on plant species and tissue specificity [47], this pathway produces diverse subsets of alcohols, aldehydes, divinyl ethers, esters, epoxides, hydroxides, hydroperoxides, ketols, ketones and triols [46,48] (Figure 3). These metabolites are implicated in nearly all plant biological processes that include defense, development and trans-kingdom communication [40,49].

In the maize B73 genome, this enzyme family is comprised of 13 members categorized into either the 9- or 13-LOX groups depending on the position the oxygen is incorporated into the fatty acid carbon backbone [50,51]. Only 13-LOXs are capable of catalyzing 13(*S*)-hydroperoxide octadecatrienoic acid (13-HPOT, (9Z,11E,13S,15Z)-13-hydroperoxyoctadeca-9,11,15-trienoic acid), from α-linolenic acid (C18:3) with appropriate regio- and stereo-specificity required for JA biosynthesis. The initial steps of JA biosynthesis (lipases, LOX, AOS and AOC) are localized in plastids, and therefore, most 13-LOXs possess plastid transit peptides (CTP) for close proximity to linolenate. 

**Figure 4:**
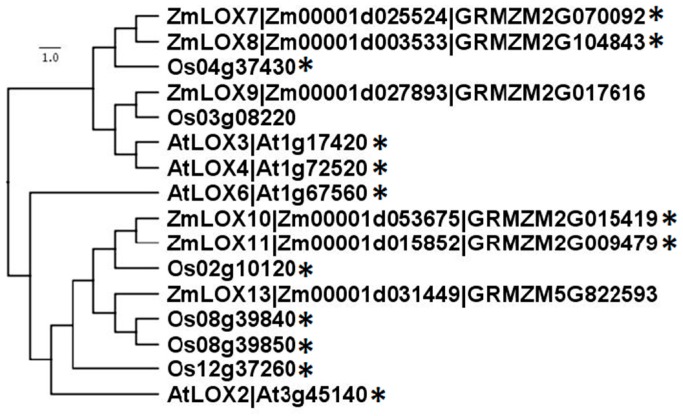
Amino acid sequences of 13-LOX family members from *Arabidopsis*, maize, and rice compared with ClustalX and visualized with Figtree. Gene labels follow established nomenclature from *Arabidopsis* [53], maize [51], and rice [52]. * denotes predicted CTP by ChloroP.

**Figure 5:**
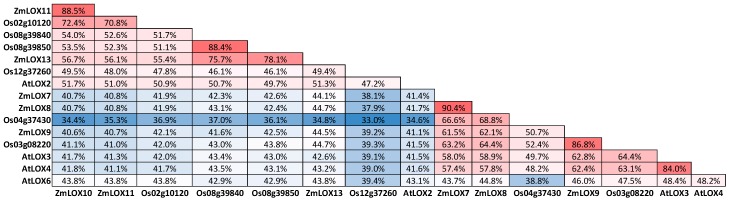
Protein sequence identity comparison of the 13-LOX family members from *Arabidopsis*, maize, and rice. Analyses was performed with Sequence Identity and Similarity software (SIAS; http://imed. med.ucm.es/Tools/sias.html). The color code (red to white to blue) scale represents the percent sequence identity from greatest to least.

Six 13-LOXs are present in the maize genome (Figure 4) and cluster into two groups. ZmLOX7 (Zm00001d025524; GRMZM2G070092), 8 (Zm00001d003533; GRMZM2G104843) and 9 (Zm00001d027893; GRMZM2G017616) cluster together and ZmLOX10 (Zm00001d053675; GRMZM2G015419), and 11 (Zm00001d015852; GRMZM2G009479) and 13 (Zm00001d031449; GRMZM5G822593) form a distinct group [51,52,53,54]. The two groups share relatively low amino acid sequence identity (Figure 5) of around 45%, yet the members of the same group share up to 90% sequence identity. While any of these 13-LOXs in maize may produce substrate for JA biosynthesis, thus far, only ZmLOX8 has been shown to be required for basal JA biosynthesis in tassel [44]. Christensen et al. (2013) have shown that ZmLOX8 is the major 13-LOX isoform in wound-induced JA biosynthesis in leaves. ZmLOX8 localization to the chloroplasts was determined by analysis of the LOX8:mCherry fluorescent protein [44]. In addition to wound-inducible JA, ZmLOX8 is essential for normal JA production during tassel primordia development, discovered in a forward genetics approach characterizing the *tasselseed1* mutant of maize [44]. ZmLOX8 shares 94% sequence identity with ZmLOX7, suggesting that ZmLOX7 may also be involved in JA biosynthesis. Another functionally-characterized maize 13-LOX, ZmLOX10, is solely responsible for green leaf volatile (GLV) biosynthesis and wound-induced JA; however, whether it is directly involved in JA production or signaling JA biosynthesis via GLVs needs to be further elucidated [51]. ZmLOX10-YFP (yellow fluorescent protein) was previously shown to not localize to chloroplasts and instead be found in small microbodies [51]; however, recent analysis with improved microscopy techniques positively confirm ZmLOX10-YFP presence in chloroplasts (M. K., unpublished data). Thus, it cannot be ruled out that ZmLOX10 provides substrate towards JA biosynthesis during stress response in a tissue- or stimulus-specific manner. A cautionary note, careful examination of the subcellular localization of ZmLOX9-YFP, did not find it to be localized to chloroplasts [55]. 

Another class of LOXs, termed dual positional LOXs, has dioxygenase activity on both the 9- and 13- carbon positions [56,57,58,59]. In maize, two dual positional LOXs exist, ZmLOX1 (Zm00001d042541; GRMZM2G156861; DQ335760) [60] and ZmLOX2 (Zm00001d042540; GRMZM2G156861 [sic]; DQ335761). Both lack a predicted plastid transit peptide sequence as determined by ChloroP [61]. Interestingly, when expressed transgenically in rice, ZmLOX1 was able to associate with chloroplast membranes in the presence of calcium [62]. A similar dual positional LOX from rice was determined to localize into chloroplasts and when silenced, failed to produce increased JA following brown plant hopper (*Nilaparvata lugens*) infestation [59]. Therefore, genetic evidence is required to definitively assign a role to the dual positional maize LOXs, ZmLOX1 and 2. Unfortunately, these two genes share 87% amino acid identity and are tandemly duplicated, suggesting functional redundancy. To completely characterize the role of maize dual positional LOXs, the creation of double mutants is necessary, but problematic. It is likely that next generation genome editing tools will be required for this endeavor [63]. In summary, the most likely candidate isoforms for JA biosynthesis are ZmLOX7, 8, 9, 10, 11 and 13. 

## 6. Allene Oxide Synthase

The 13-LOX product 13-HPOT is catalyzed into an epoxide, 12,13(*S*)-epoxy-octadecatrienoic acid, by 13-AOS. 13-AOS, along with the closely related 9-AOS, hydroperoxide lyase (HPL) and divinyl ether synthase (DES), belong to the cytochromeP450 monooxygenase (CYP) enzyme family and part of the CYP74 group (Figure 3) [64]. The CYP74s are involved in the metabolism of the immediate LOX products, hydroperoxy fatty acids. All HPLs, DESs and AOSs utilize the same substrate, but form distinct products, and the enzymes cluster into different clades. The CYP74A clade contains all 9-, 9/13- and 13-AOSs. 13-AOS serves as the first committed enzymatic step for the production of JA [65]. Interestingly, these enzymes are extremely similar, and even a single amino acid substitution repurposed the AOS from *Arabidopsis* into a functional HPL [66]. 

**Figure 6:**
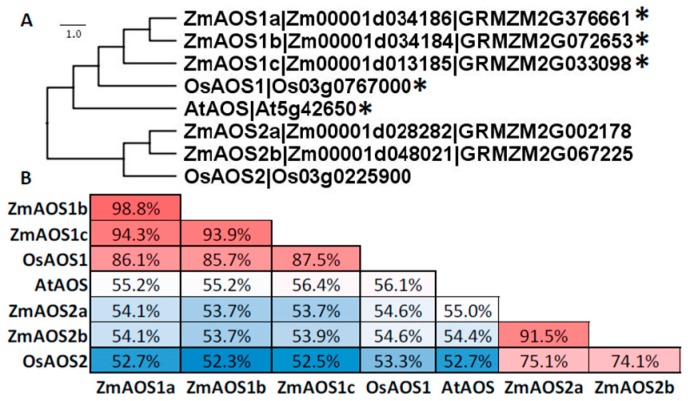
Amino acid sequences of CYP74A family members from *Arabidopsis*, maize, and rice compared with ClustalX and visualized with Figtree. (**A**) Gene labels follow nomenclature for maize (this chapter) and rice [72]. * denotes predicted CTP by ChloroP. (**B**) Identity analyses was performed with SIAS. The color code (red to white to blue) scale represents the percent sequence identity from greatest to least.

Previous research reported three maize AOS isoforms [67], but after analysis of the maize B73 genome, five AOSs were identified (Figure 6). These five isoforms cluster into two clades, termed here AOS1 and 2. AOS1 clade contains ZmAOS1a (Zm00001d034186; GRMZM2G376661), b (Zm00001d034184; GRMZM2G07265) and c (Zm00001d013185; GRMZM2G033098), and AOS2 contains ZmAOS2a (Zm00001d028282; GRMZM2G002178) and b (Zm00001d048021; GRMZM2G067225). Currently, no specific 13-AOS has been characterized in maize. Nearly all JA-producing 13-AOSs are localized in plastids and, similarly to 13-LOXs, encode CTP sequences with few exceptions, a notable example being the AOS from barley (*Hordeum vulgare*) [68]. The three maize AOS1s contain predicted CTPs [61] that are expected to target them to plastids. The ZmAOS1 clade members also cluster with OsAOS1 at ~86% sequence identity. OsAOS1 was shown to contain a CTP and identified through a preliminary mapping of a mutant in phytochrome-mediated inhibition of *coleoptile photomorphogenesis growth* (*cpm1*) [69] that was later determined to be NPH3 (*NONPHOTOTROPIC HYPOCOTYL3*) [70]. Two previously predicted rice AOSs were later determined to encode for HPLs and have been appropriately renamed [71] and are not included in this analysis. 

Similar to the dual positional specific LOXs of maize [62] and rice [59], the barley AOS [68], rice AOS2 (Os03g0225900) [73] and recently maize (ZmAOS2b) [74] were reported to possess dual substrate specificity capabilities of catalyzing either 9- or 13-HPOD/T into the corresponding allene oxides. None of the AOS2 clade members (Figure 6) encode predicted CTPs. Recombinant ZmAOS2b was determined to catalyze 9-HPOD into 9,10-epoxy-10,12-octadecadienoic acid (9,10-EOD). In that study, maize root extract possessed a cyclase activity to convert 9,10-EOD into (9*S*,13*S*)-10-oxo-phytoenoic acid (10-OPEA). In rice, while attempting to produce male-sterile plants with an RNAi approach, silencing OsAOS2 was determined to be more effective than silencing OsAOS1 [75]. This observation suggests that either members of the AOS2 clade produce JA directly due to their dual substrate specificity or function through the production of a yet to be determined oxylipin signal that promotes JA-mediated male fertility. In agreement with this hypothesis, a 9-LOX mutant of maize had reduced JA content [76].

Very few studies have explored expressional analysis of the AOS1 clade [67]. It is important to bring to attention that most published reports analyze the expression of the maize AOS2 clade [45,77,78,79,80,81]. These data should be treated with caution until genetic evidence reveals which of the AOS isoforms are directly involved in JA production in maize.

## 7. Allene Oxide Cyclase

Cyclization of 12,13(*S*)-EOT into stereospecific (9*S*,13*S*)-12-oxo-phytodienoic acid (12-OPDA) occurs via allene oxide cyclase (AOC) and represents the first enzymatic step to yield a member of the jasmonate family of oxylipins, 12-OPDA. It is predicated that instead of directly catalyzing the reaction, AOC constrains 12,13(*S*)-EOT into the appropriate configuration for self-cyclization in a stereospecific manner [82,83]. 

**Figure 7:**
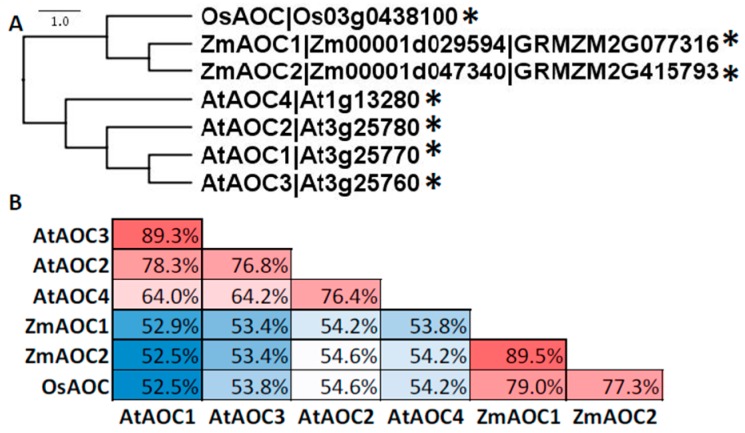
Amino acid sequences of AOC family members from *Arabidopsis*, maize, and rice compared with ClustalX and visualized with Figtree. (**A**) Gene labels follow nomenclature from *Arabidopsis* [84], maize (this chapter), and rice [72]. * denotes predicted CTP by ChloroP. (**B**) Identity analyses was performed with SIAS. The color code (red to white to blue) scale represents the percent sequence identity from greatest to least.

The maize genome encodes two AOC isoforms, which are termed here ZmAOC1 (Zm00001d029594; GRMZM2G077 316) and ZmAOC2 (Zm00001d047340; GRMZM2G415793) and cluster closer with the sole rice AOC (Figure 7) [72] (~78% sequence identity) than with the four AOCs from *Arabidopsis* [84]. The availability of an additional AOC in maize compared to rice may convey functional redundancy, but also provide a tissue- and stress-specific regulation, as was observed for the tissue- and promoter-activity of the four AOC isoforms in *Arabidopsis* [84]. A maize AOC isolated from seed was shown to utilize only 18:3-derived, but not 18:2-derived 12,13-epoxy fatty acids; however, the identity of this AOC has not yet been determined [85]. While in maize no functional analysis of AOC have been reported, in rice, the JA-deficient *hebiba* (Japanese for “snake leaf”) mutation [86] and *coleoptile photomorphogenesis2* (*cpm2*) [72] mutants lack the sole AOC. Recently, it was determined that in addition to missing AOC, *hebiba* is also deleted in DWARF14-like [87], which imparts defects root interactions with arbuscular mycorrhize through the karrikin (the so-called, “smoke hormone”) receptor [88] and 25 other genes [89]. Because of the complicated nature of the *hebiba* mutation, the function of JA in rice will need to be revisited by using JA-deficient mutants disrupted in single genes. Interestingly, *cpm2* and *hebiba* displayed increased tolerance to salt [90], but it remains to be elucidated whether JA-deficiency in maize impacts salt tolerance. Given that the two maize AOCs share ~90% sequence identity, it is reasonable to expect functional redundancy will require double knockout mutants to be used to establish their role in JA synthesis. Fortunately, ZmAOC1 and 2 are located on chromosomes 1 and 9, respectively, so the double mutant can be generated through conventional breeding.

## 8. Oxo-Phytodienoic Acid Reductase and Beta-Oxidation

Reduction of 12-OPDA occurs in the peroxisome via Type II 12-OPDA reductases (OPRs). Type II OPRs reduce the cyclopentenone *cis*-(+)-12-OPDA into the cyclopentanone, OPC-8:0 (8-[3-oxo-2-*cis*-[(*Z*)-2-pentenylcyclopentyl]octanoic acid). 

**Figure 8:**
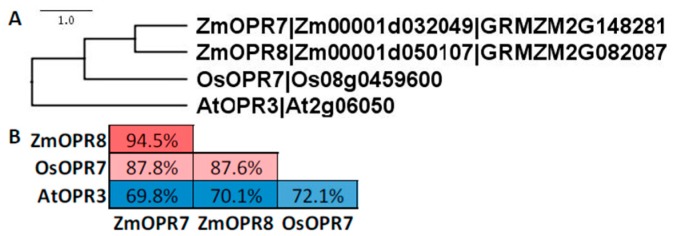
Amino acid sequences of Type II OPR family members from *Arabidopsis*, maize, and rice compared with ClustalX and visualized with Figtree. (**A**) Gene labels follow nomenclature from maize [91] and rice [92]. (**B**) Identity analyses was performed with SIAS. The color code (red to white to blue) scale represents the percent sequence identity from greatest to least.

The maize genome encodes eight OPR isoforms, yet only two are Type II OPRs [91], ZmOPR7 (Zm00001d032049; GRMZM2G148281) and ZmOPR8 (Zm00001d050107; GRMZM2G082087). ZmOPR7 and 8 share ~87% and ~70% sequence identity, respectively, with rice [92] and *Arabidopsis* (Figure 8) [93,94] JA-producing OPRs. In the B73 inbred line, *ZmOPR7* and/or *ZmOPR8* are induced by JA, ethylene and abscisic acid in leaves [91]. OPR7 and OPR8 share ~95% sequence identity, and recent functional characterization showed that ZmOPR7 and 8 are functionally redundant and both are capable of reducing 12-OPDA [45]. Supporting the case of redundancy for the two genes in JA biosynthesis, only *opr7opr8* double mutants, but not single mutants displayed reduced JA [45]. As expected, 12-OPDA levels of *opr7opr8* double mutants were similar to WT. To date, the *opr7opr8* double mutant provides the best genetic evidence for the role of JA in maize. The phenotypes of the double mutant are described in detail below. 

Three rounds of beta-oxidation are required to shorten the octanoic acid side change of OPC-8:0 to ethanoic acid. The three rounds of beta-oxidation are performed by three distinct enzymes, ACX (acylcoenzyme A (CoA) oxidases), MFP (multifunctional proteins), and KAT (3-ketoacyl-CoA thiolases) [32]. The product of the three reactions is JA.

## 9. JA-Amino Acid Conjugation

Many chemically-diverse jasmonic acid derivatives were reported from different plant species [33]. In summary, JA can be converted to one of its derivatives by conjugation with a number of amino acids, decarboxylation, glycosylation, hydroxylation of a number of carbon positions, autocyclization of hydroxy-JAs into lactones, methylation, sulfonation or by more than one modification. The conjugation of JA with the ethylene precursor, ACC (1-aminocyclopropane-1-carboxylic acid), has also been reported [95]. JAs were also found with an unsaturated cyclopentenone (didehydro-JAs), with a saturated pentanyl moiety (dihydro-JAs) or with its ethanoic acid chain elongated into three (homo-JA) or four carbons (dihomo-JA) [96]. In addition, JA is capable of undergoing stereo-rearrangement of its ethanonic acid and pentenyl chains and can be found in the 3*R*, 7*R* or 3*R*, 7*S* (7-*iso*-JA) configurations. These different jasmonates possess different effects on JA-inducible gene expression, and while most have shown no discernible activity, several were able to induce or suppress JA-responsive genes [97]. Recently, (+)-7-*iso*-jasmonoyl-l-isoleucine (JA-Ile) was determined to be the most effective ligand for the SCF(COI1) receptor [98], currently the only JA receptor identified in plants.

**Figure 9:**
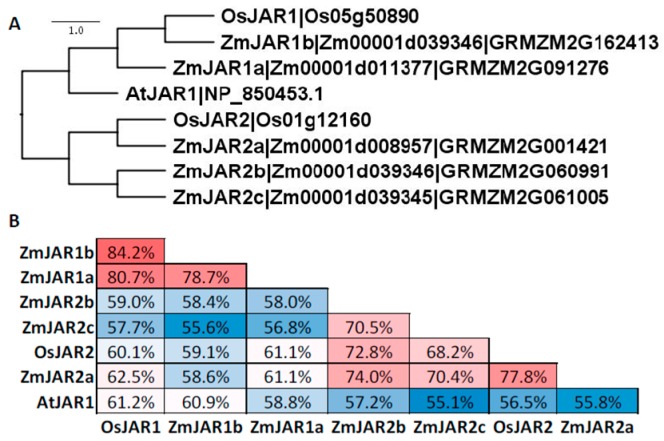
Amino acid sequences of JAR1-like family members from *Arabidopsis*, maize, and rice compared with ClustalX and visualized with Figtree. (**A**) Gene labels follow nomenclature from maize (this chapter) and rice [100]. (**B**) Identity analyses was performed with SIAS. The color code (red to white to blue) scale represents the percent sequence identity from greatest to least.

The conjugation of JA with Ile occurs through the JAR1 (JASMONATE RESISTANT1) enzyme [99]. The JAR1 enzyme belongs to the large firefly luciferase gene family. In *Arabidopsis*, only one protein, AtJAR1 was shown to utilize JA as a substrate [99]. In rice, two proteins were shown to produce JA-Ile in vitro, OsJAR1 and OsJAR2 [100]. The maize genome encodes five JAR1-like isoforms that group into two clusters (Figure 9). The JAR1 cluster contains ZmJAR1a (Zm00001d011377; GRMZM2G09127) and ZmJAR1b (Zm00001d039346; GRMZM2G162413), which share ~60% sequence identity with AtJAR1. The JAR2 cluster contains ZmJAR2a (Zm00001d008957; GRMZM2G001421), ZmJAR2b (Zm00001d039346|GRMZM2G060991) and ZmJAR2c (Zm00001d039345; GRMZM2G061005). These isoforms only share ~56% sequence identity with AtJAR1, but 68 to 78% sequence identity with OsJAR2. 

To date, no maize JAR1-like isoform has been characterized. In rice, JAR1 was determined to be involved in photomorphogenesis [101], floret development [102] and phytoalexin production [103]. The presence of multiple JAR1-like isoforms in maize will require utilization of double mutants for functional characterization. 

## 10. 9-Oxylipin Jasmonate Analogs (10-OPEA, 10-OPDA and Derivatives)

In addition to the 13-LOX-derived jasmonates, plants possess a parallel pathway involving 9-LOX reactions that also produce cyclopenta(e)nones, analogous to JAs [67]. These 9-LOX products, 10-oxo-11-phytoenoic acid (10-OPEA) and 10-oxo-11,15-phytodienoic acid (10-OPDA), are structurally related to 12-OPDA and first described in potato [104]. The 9-LOX isoforms responsible for their biosynthesis have not been identified yet in maize or any other plant species. In maize, following 9-LOX activity on C18:2, the 9-hydroperoxy fatty acid (9-HPOD) is catalyzed into 9,10-epoxy octadecadienoic acid (9,10-EOD) through the action of ZmAOS2b [74]. The 9,10-EOD is cyclized into 10-OPEA by an, as yet uncharacterized, AOC-like enzyme [74]. 

Recently, these cyclopentanones were described to possess potent signaling, insecticidal and antimicrobial activities in maize [67,105]. Transcriptional analysis uncovered that JA, 12-OPDA or 10-OPEA regulate unique subsets of genes, suggesting distinct signaling activities [67]. In wounded silk and root tissue, 10-OPEA accumulates as the predominant cyclopentanone species followed by 10-OPDA [105]. Low concentrations of 10-OPEA added to artificial diet media reduce the growth of *Helicoverpa zea* (corn earworm) by half compared to insects grown on control media. 10-OPEA and its derivatives accumulated in local tissue following inoculation by *Cochliobolus heterostrophus*, the causal agent of southern corn leaf blight, to greater levels than either 12-OPDA or JA. While not having direct antimicrobial effects against *C. heterostrophus*, concentrations as low as 170 μM reduced growth of *Fusarium verticillioides* and *Aspergillus flavus*. Direct application of 10 μL of 2 mM of 10-OPEA caused dramatic phytotoxicity due to programmed cell death (PCD). Most surprisingly, 10-OPEA promoted the greatest necrosis and ion leakage compared to all other PCD-promoting compounds tested, including fumonisin B1 [67].

## 11. Regulation of JA Production by the Non-JA Producing Branches of the LOX Pathway

### 11.1. The Role of GLV-Mediated Signaling on JA Production

In maize, ZmLOX10 was determined to serve as the sole 13-LOX isoform responsible for basal, herbivore- and wound-induced GLV production in aboveground tissue [51]. Following ZmLOX10 activity, the C18:2- or C18:3-derived 13-hydroperoxy fatty acids are cleaved into C6 aldehydes and C12-oxo-fatty acids, via the action of hydroperoxide lyase (HPL) [106]. The C6 aldehydes can be subsequently converted into C6 alcohols, acetylated or hydroxylated [107]. Transposon-disrupted *lox10* knockout mutant plants are completely devoid of GLVs and were utilized as a genetic tool to understand the role of GLVs in regulation of JA production. Compared to wild-type (WT) plants, *lox10* mutants accumulated significantly lower levels of 12-OPDA and JA in locally-damaged tissue after wounding treatment compared to WT. The *lox10* mutants also showed reduced transcript levels of the JA biosynthesis genes, *ZmLOX8* and *ZmOPR7/8*, suggesting that GLVs are required for normal wound-induced JA via transcriptional regulation.

### 11.2. The Role of 9-LOX-Mediated Signaling on JA Production

Recent evidence suggests that 9-oxylipins regulate maize JA production, in both a positive or negative manner. ZmLOX3 is a 9-LOX and has been shown to serve as a negative regulator of JA biosynthesis in roots and seed. The *lox3* knockout mutant roots produced elevated basal levels of JA and displayed an increased expression of JA biosynthesis genes [77], suggesting that LOX3-produced 9-oxylipins suppress the production of JA. Interestingly, transcript levels for GLV-biosynthesis genes, Zm*LOX10* and *ZmHPL*, were also elevated in untreated *lox3* roots. In seeds infected by *A. flavus*, *lox3* mutant accumulated increased levels of linolenic acid and JA compared to WT. These studies suggest that ZmLOX3 suppresses JA biosynthesis in diverse organs. In contrast to ZmLOX3, another predominant 9-LOX, ZmLOX12 appears to function as a positive regulator for JA production [76]. In response to *F. verticillioides* infection, *lox12* knockout mutants were unable to induce accumulation of 12-OPDA, JA and JA-Ile. The decrease in JAs was accompanied with decreased transcript accumulation of JA biosynthetic genes and increased susceptibility of *lox12* mutants to *F. verticillioides*. Taken together, these reports suggest that 9-LOX-derived oxylipins help fine-tune JA biosynthesis in organ- and stress-inducible fashion.

## 12. JA in Maize Defense against Insects

### 12.1. Plant Defenses, Genetic Evidence and Insect Elicitors

Plant defenses against insects are classified according to nature (physical or chemical), mode of action (direct or indirect) and timing (constitutive or inducible) [108]. Physical defenses are anatomical structures that provide increased durability to herbivory. Chemical defenses utilize toxins or other molecules to deter feeding or nutrition acquisition. Direct defenses explicitly target the insect herbivore physiology and survival, while indirect defenses rely on the attraction of predators of the herbivores. Constitutive defenses are preformed and persist through herbivory, while inducible defenses are activated only during the perception of an herbivore or herbivory-associated tissue damage. While the role of JA in dicot insect defense has been well studied [109], especially in tobacco and tomato, there is limited information on the role of JA in maize insect defenses. 

As in other studied plants, JA in maize is critical to successfully thwarting insect attack. The strongest evidence for the role of JA in maize insect defense stems from studies using the JA-deficient *opr7opr8* double mutants’ response to herbivory by beet armyworm (*Spodoptera exigua*) [45]. Double mutants were consumed by beet armyworm more readily than WT, which was accompanied with an increased larval weight. The significance of wound-induced JA in insect defenses was also confirmed by the analysis of the *lox10* mutant that was unable to induce accumulation of JA in response to wounding [51]. Similar to *opr7opr8*, mutant seedlings of *lox10* lost more mass due to insect feeding and supported greater larval weight gain compared to WT. 

As in other plants, JA serves as a major regulator for diverse insect resistance mechanisms [110], but its precise mode of action in maize is still under investigation. Here, we present recently published studies highlighting the impact of JA on diverse lines of maize defenses against herbivory. In maize, JA biosynthesis in response to insect feeding has been studied extensively using insect elicitors. Of the insect-derived elicitors described so far, volicitin, known chemically as *N*-(17-hydroxylinolenoyl)-l-glutamine (17-OH-C18:3-Gln) [111] and *N*-linolenoyl-glutamine (C18:3-Gln) [112], possesses the greatest activity on maize JA activation [113]. An application of either elicitor caused JA accumulation to spike after treatment. Volicitin, crude regurgitant and mechanical wounding increased local JA concentration when applied to leaf tissue, but only the treatments by crude regurgitant or volicitin induced the accumulation of JA in the distal portion of the leaf [78]. In terms of basal tissue, treatment with volicitin was unable to induce JA accumulation after application [78]. However, treatment with C18:3-Gln induced expression of *ZmMYC7E*, a putative orthologue of the *Arabidopsis* transcription factor AtMYC2, in maize (Zm00001d030028; GRMZM2G001930), after application in both basal tissue and in untreated systemic leaves [80].

### 12.2. Direct Defenses

One type of direct physical defense is trichomes. Trichomes obstruct insects and are capable of storing toxic defensive metabolites that are released upon damage. JA positively regulates trichome formation. Exogenous application of JA on maize leaves induced the development of trichomes [114]. Another physical defense is lignification, which strengthens cell walls to increase resistance against insect mastication. Recently, treatment with MeJA resulted in degradation of repressors of the lignin biosynthetic pathway [115]. Direct chemical defense responses include the production of metabolites that intoxicate or repel herbivores or suppress nutrient acquisition (e.g., protease inhibitors). Benzoxazinoids are indole derived hydroxamic acids (e.g., DIMBOA, 2,4-dihydroxy-7-methoxy-1,4-benzoxazin-3-one) that can have direct insecticidal activity [116]. ZmIGL (INDOLE-3-GLYCEROL PHOSPHATE LYASE) is capable of generating free indole for benzoxazinoid biosynthesis and is inducible by MeJA application [117]. Similarly, application of JA induced accumulation of 2-(2-hydroxy-4,7-dimethoxy-1,4-benzoxazin-3-one)-β-d-glucopyranose (HDMBOA-Glc) in levels similar to insect herbivory [118].

Several recent studies have identified potential mechanisms of JA-mediated herbivory defense in maize. For example, a new class of diterpenoids derived from *ent*-kaurane [119] (precursor for gibberellic acid) was increased upon insect feeding when leaves were pre-treated with a combination of JA and ethylene [120]. Kauralexin A3 or B3 treatment on maize stems reduced feeding by European corn borer (*Ostrinia nubilalis*) [120]; however, kauralexins may not have direct insecticidal activity, as artificial diet enriched with high concentrations failed to affect *O. nubilalis* growth [119]. 

Cysteine proteases are defensive enzymes produced by plants, which when consumed by insects damage their gut [121]. In maize, the best-characterized defensive cysteine protease is ZmMIR1 (MAIZE INSECT RESISTANCE1, Zm00001d036542; GRMZM2G150276) [122,123]. In the place of JA, another phytohormone, ethylene (ET) appears to be the major regulator of ZmMIR1. Treatment with 2-chloroethylenphosphonic acid (CEP), an ET-releasing chemical, was able to induce *ZmMIR1* transcript accumulation more dramatically than MeJA treatment [124]. Application of an ET inhibitor reduced aphid-induced *ZmMIR1* expression and improved aphid performance [125]. Plant protease inhibitors inactivate insect digestive enzymes [126] and slows their growth and development [127]. In several dicot species, including tomato, tobacco and *Arabidopsis*, JA is the major hormone mediating the induction of protease inhibitor production [128,129]. Similarity, *ZmMPI* (MAIZE PROTEASE INHIBITOR, Zm00001d011080, GRMZM2G028393) is induced more strongly by MeJA treatment than by CEP [124] and is upregulated in *lox3* knockout mutant roots, overproducers of JA [77]. *ZmMPI1* is clearly JA-dependent, as induction of this gene by wounding is significantly reduced in the JA-deficient *opr7opr8* double mutants. In addition to protease inhibitors that inactivate native insect digestive proteins, plants are capable of producing non-protein amino acids that are incorrectly incorporated into protein synthesis. MeJA transiently induced the production of 5-hydroxynorvaline, and aphids fed diets supplemented with 5-hydroxynorvaline at physiological concentrations suppressed aphid reproduction [130]. 

In addition to signaling for resistance, JAs can display direct toxicity on insects in a manner similar to what was described for their 9-LOX analogues [67]. The α,β-unsaturated carbonyl structure makes a subset of jasmonate (e.g., 12-OPDA) reactive electrophilic species (RES) and subsequently toxic metabolites. To circumvent this toxicity, ear- and army-worms possess isomerases that can rearrange the double bond in the cyclopentenone ring to disrupt the α,β-unsaturated carbonyl structure, which detoxifies 12-OPDA [131]. It was shown that silencing of an isomerase gene involved in the 12-OPDA detoxification reduces the insect performance [132].

### 12.3. Indirect Defenses

Indirect defense is another strategy of plants to resist insect herbivory, and it relies on the emission of volatile organic compounds (VOCs) to recruit beneficial predators of insect herbivores [133]. Following insect damage, JA accumulation was positively associated with increased indole and sesquiterpene emissions [134] and was correlated with the site of terpene release [135]. While mechanical damage and volicitin induced similar levels of JA in intact or excised leaves, treatment with either JA or volicitin results in a greater release of sesquiterpenes from excised leaves compared to intact leaves [136]. Additionally, ethylene enhanced volatile emission from volicitin and JA treatment, but not mechanical damage. Treatment with JA increased the attractiveness of maize plants to the parasitoid wasp, *Cotesia kariyai* [137]; however, JA did not interfere with the ability of *C. kariyai* to discriminate between armyworm (*Mythimna separata*) infested and uninfested maize plants. Interestingly, application of both JA and a derivative of a JA precursor, methyl-C18:3, produced a comparable increase of *C. kariyai* attraction to maize plants [138]. However, the volatile profiles emitted from JA or methyl-C18:3 were substantially different. Methanol and the GLV, Z-3-hexenyl acetate, were found to be the most attractive volatiles, respectively, from the methyl-C18:3 or JA treatment. It remains to be seen if these observations can be repeated under natural conditions [139]. 

Another indirect defensive role of VOCs is their ability to induce intra- and inter-plant defenses in a phenomenon known as priming. The priming of plants is the ability to activate defenses earlier and to a greater level after exposure to a stimulus [140]. One such stimulus is the volatile blend released from an injured neighbor or by exogenous volatile treatment. The effect of the priming response is determined by subjecting the exposed plants to stress such as wounding or herbivory after 24 h of priming treatment and monitoring JA accumulation or VOC emissions. The priming state persists for at least five days following initial treatment [141] and is likely reliant on chromatin modification to prepare the transcriptional machinery to activate defense genes similar to what is observed with systemic acquired resistance (SAR) against pathogens [142]. JA accumulation is responsive to the volatile-mediated priming. Maize plants pretreated overnight with GLVs facilitated enhanced JA induction following regurgitant application compared with non-pretreated plants [143]. Similarly, a subset of JA-responsive defensive genes also displays a priming pattern in response to airborne volatiles [144].

While GLVs [143], indole [145] and other VOCs [144] are better understood in their role in priming, much less is known concerning the role of jasmonates during priming activation. JA itself can be converted into several volatiles that serve roles in the priming response. One example of a JA-derived volatile capable of inducing a priming response is the decarboxylated JA derivative, *cis*-jasmone. When cis-jasmone is pre-applied to maize plants and incubated for 24 h, treated plants produced greater VOC emissions rapidly after leafhopper (*Cicadulina storeyi*) infestation [146]. Treatment with another volatile JA derivative, MeJA, induces *ZmIGL* [117], thus potentially indirectly signaling for indole-mediated priming [145]. Curiously, multiple tests in several laboratories, including the groups of Engelberth and Kolomiets, have failed to detect any measurable amounts of MeJA from maize, suggesting that the inbred lines that were tested do not produce MeJA. It remains to be seen if other maize lines or teosintes produce this volatile jasmonate.

## 13. JA in Maize Pathogen and Nematode Defense

JA-mediated defenses are the principle countermeasures against pathogens with a necrotrophic lifestyle [147]. In maize, JA-deficient *opr7opr8* mutants are not capable of surviving under field conditions due to “damping-off” disease [45]. In that study, the major pathogen responsible for “damping-off” in the diseased roots of *opr7opr8* was identified to be *Pythium aristosporum*. Supporting the genetic evidence that maize requires JA for immunity against *P. aristosporum*, exogenous application of JA as a soil drench could rescue normal survival of the plants. Any study with *opr7opr8* double mutants requires the use of sterile soil to exclude the plants from soil-borne pathogens. JA is also required for immunity against another common opportunistic soil-borne fungus, *F. verticillioides*, as the *opr7opr8* mutants succumb to mesocotyl inoculation as rapidly as 8 h post-infection by this pathogen [76]. In another study, JA accumulation and JA biosynthetic gene induction correlated with increased resistance against *F. verticillioides* stalk rot in a CO_2_-dependent manner [148]. A recent genome-wide association study of maize infected by *A. flavus* identified the JA pathway as the most impacted between resistant and susceptible genotypes [149]. To date, however, no genetic evidence was reported that clearly determines the role of JA in either defense or potentially even benefiting the pathogenicity of this important mycotoxin-producing fungus. In this regard, it is worthy to note that the *lox3* knockout mutant was more susceptible to *A. flavus* and possessed increased JA accumulation in response to infection by this pathogen [150]. To unambiguously establish the role of JA in maize interactions with *A. flavus*, the utilization of JA-deficient *opr7opr8* [45] will be required. The male infertility defect of this double mutant will make this analysis technically challenging, as this pathogen is a seed resident. 

A potential mechanism for JA-mediated signaling in response to fungal pathogens is through the peptide signal, ZmPEP1 [151]. In a pattern reminiscent of the interplay between the peptide hormone, systemin and JA in tomato [152], exogenous ZmPEP1 and JA application induce each other’s accumulation. ZmPEP1 treatment also induces HDMBOA-Glc accumulation and benzoxazinoid biosynthetic genes. Pretreatment with ZmPEP1 increased resistance to fungal pathogens, *C. heterostrophus* and *Colletotrichum graminicola*.

In contrast to the role of JA in defense, JA may also facilitate increased pathogenesis. Elevated endogenous JA correlates with increased susceptibility to root-knot nematodes (*Meloidogyne incognita*) infestation [77]. The *lox3* mutant maize line, disrupted in the predominantly root-expressed 9-LOX, possessed more JA content in roots compared with WT maize seedlings [77]. Furthermore, *lox3* mutant seedlings supported more root-knot nematode eggs compared to WT seedlings. Additionally, *lox3* mutants were more attractive to *M. incognita* juveniles than WT as determined by a six-arm root olfactometer choice assay The elevated JA content corresponded to increased expression of the JA biosynthetic genes, as well as the 10-OPEA producing *ZmAOS2b*, suggesting the suppression of the JA pathway at the transcriptional level via ZmLOX3-mediated signaling. It will be of interest to determine the role of 9-LOX-derived cyclopenta(e)nones during maize-nematode interactions. A definitive role of JA in susceptibility to nematodes will require the creation of triple mutant *lox3opr7opr8*, but because of male sterility and increased susceptibility of JA-deficient *opr7opr8* to *Pythium*, it will be difficult to maintain.

## 14. JA in Maize Symbiosis

Colonization of roots by beneficial microorganisms can instill beneficial traits upon the plant host [153]. One of the most intriguing traits is induced systemic resistance (ISR) that primes the plant for increased resistance against pathogens. This phenomenon is believed to rely on JA and ET-mediated signaling in studied dicot species, but relatively little is known about the role of JA in maize symbiosis compared to other plants. A JA role in the positive regulation of ISR in maize came from a recent report showing that the JA-overproducing *lox3* mutant display constitutively activated ISR effective against multiple foliar and stem pathogens due to over production of as-yet unidentified long distance signals found in xylem-enriched exudates [154]. Because *lox3* mutant roots constitutively overexpress all of the JA biosynthesis genes and produce elevated levels of JA in roots [77], it is likely that JA and/or other jasmonates are responsible for the constitutively-active ISR displayed by *lox3* mutant. Additional evidence for JA’s role in ISR signaling was obtained from the study of maize ISR responses conferred by root colonization with bacterial symbiote *Pseudomonas putida*. Maize roots inoculated with this beneficial bacterium were characterized by increased expression of jasmonic acid biosynthesis genes and increased resistance against foliar pathogen, *C. graminicola* [155]. Interestingly, priming by *P. putida* produced greater emissions of JA-induced aromatic volatiles and terpenes [156].

## 15. JA in Maize Growth, Development and Senescence

The strongest evidence for the role of JA in maize growth, development and senescence came from the functional analyses of JA-deficient *opr7opr8* double mutants. *opr7opr8* mutants are defective of total JA accumulation in young tissue [45]. Fitting with the role of JA in negatively regulating plant vegetative growth [157], seedlings of *opr7opr8* double mutants possess long coleoptiles, first leaf sheathes and first and second leaf blades that displayed increased growth compared with WT seedling tissues [158]. In addition to the increase growth in leaf-associated tissue seen in *opr7opr8* mutants, an increase in root lateral root length and density was also detected [158]. 

The most striking developmental effect of JA in maize is on the formation of the male reproductive organ called the tassel [44,45]. Lack of JA in developing tassels of *lox8* mutants (*tasselseed1*, *ts-1*) [44] or *opr7opr8* mutants failed to abort pistils in the developing florets [159], resulting in the so-called *tasselseed* mutant phenotype. While these mutants are capable of setting viable seeds when cross pollinated, they are completely male sterile. Exogenous JA application was able to rescue the defective masculinization of the male florets in both *lox8* single [44], *opr7opr8* double mutants [45] and another related gene, *tasselseed2* (*ts2*) [44]. TS2 was determined to encode a short-chain alcohol dehydrogenase required for normal tassel masculinization, as well [160]. Subsequent reports list TS2 as a 3β/17β-hydroxysteroid dehydrogenase [161], with implications in linking JA-mediated signaling with brassinosteroid-dependent tassel masculinization [162]. Unlike the mutation of the LOX8 gene, which impacts JA production in the tassel, but not in other organs, likely due to redundancy in JA producing LOXs (i.e., LOX7 and 9), *opr7opr8* mutants are completely devoid of JA in many other organs and possess additional reproductive developmental defects relating to the female sexual organ [45], known as the ear. While WT maize plants only develop one to two ears upon sexual maturity, *opr7opr8* double mutants initiate ear axillary buds at nearly every internode along the maize stem. Furthermore, developing ears of *opr7opr8* possess extremely elongated ear shoots three- to four-times the length of WT. Similar to the chemical complementation rescue of normal tassel, treatment with JA successfully restored the aberrant female sexual organ phenotype to normal.

Normal JA levels accelerate maize leaf senescence. The first and second leaf of double mutant *opr7opr8* seedlings had an increased lifespan compared to WT leaves. The senescence delay correlated with decreased ethylene emissions, *cis*-zeatin 9-riboside production and abscisic acid accumulation in *opr7opr8* double mutants compared with WT [45].

## 16. JA in Maize Anthocyanin Pigmentation and Photomorphogenesis

JA signaling is necessary for anthocyanin pigmentation of maize brace roots and leaf auricle [45]. While brace roots and the leaf auricle of WT, *opr7* and *opr8* single mutants displayed a normal deep red pigmentation due to prominent anthocyanin accumulation, brace roots of *opr7opr8* did not display any anthocyanin pigmentation and remained a yellow-green color [45]. The depigmented phenotype of *opr7opr8* was restored by exogenous JA application. The lack of anthocyanin correlated with a decrease in expression of two anthocyanin biosynthetic genes, *FLAVANONE 3-HYDROXYLASE* (*F3H*) and *DIHYDROFLAVONOL REDUCTASE* (*A1*). Both genes had nearly an eight-fold decrease in expression in *opr7opr8* double mutants compared with WT levels [45]. JA is required for maize seedlings to experience normal light-induced development. When germinated and grown in darkness for seven days, *opr7opr8* double mutants grew longer mesocotyls and coleoptiles compared with WT, indicative of exaggerated skotomorphogenesis [158]. However, when grown under continuous red light (an external signal for photomorphogenesis) and compared to WT, *opr7opr8* double mutant continued to display longer mesocotyl and coleoptiles.

## 17. Maize Genes Regulated by JA

The best-characterized JA-dependent wound-responsive maize genes were determined from a macroarray analysis comparing gene expression of *opr7opr8* double mutant seedling leaves to WT following mechanical damage [45]. Table 1 shows the genes that show clear dependence on intact JA biosynthesis. We recommended the use of these genes as molecular markers for JA responsive transcription. Primer sequences are available for probe construction or for semi-quantitative reverse transcription polymerase chain reaction (semi-q RT-PCR) [45]. Primer sequences for qPCR analysis are available for the lipoxygenase genes [67].

## 18. Conclusions

Given that monocots contribute, either directly or indirectly, to a substantial proportion of calories for humans and the growing interests in utilizing monocots as a renewable fuel alternative, it is crucial to improve monocot yield through environmentally-conscientious practices. Fine-tuning of phytohormone signaling can provide an avenue for this improvement, but many more studies are required to provide a comprehensive understanding of hormone biology in monocots. Generating a level of understanding comparable to dicot models for the biosynthesis, signaling and function of jasmonates in maize (and other monocots) will require a coalition of multiple research teams with diverse expertise. While not covered in this review, recent studies with *Arabidopsis* suggests that various jasmonates and their derivatives have their own independent roles in multiple physiological processes [15,157,158]. Additionally, many of these JA derivatizations are under enzymatic control, and orthologous for the genes involved are found in the maize genome. Together, this suggests that plant responses to JA are under a tight regulation via conversion of one jasmonate species to another. Another point that requires more investigation is the cross-talk between 9-oxylipins and JA production. This complexity that is emerging in JA biology resembles the intricacies found in mammalian systems. It is reasonable to expect that in the near future, other plant oxylipins will take their place alongside JA as recognized phytohormones.

## Figures and Tables

**Table 1:** JA-dependent wound-responsive genes, adapted from [45].

Gene	Gene annotation	Gramene ID ^†^	GRMZM ID ^†^	Genbank ^†^ or TIGR ID ^†^
α,β-Unsaturated carbonyl detoxification				
ZmOPR3	12-oxo-phytodienoic acid reductase 3	Zm00001d037182	GRMZM2G156712	AY921640
ZmOPR4	12-oxo-phytodienoic acid reductase 4	Zm00001d013493	N/A in RefGen_v3	AY921641
ZmOPR5	12-oxo-phytodienoic acid reductase 5	Zm00001d003584	GRMZM2G087192	AY921642
Direct defense				
ZmMIR1	maize insect resistance 1 (mir1) cysteine proteinase	Zm00001d036542	GRMZM2G150276	NM_001112101
ZmMPI	proteinase inhibitor	Zm00001d011080	GRMZM2G028393	X78988
Ethylene associated				
ZmACS2	1-aminocyclopropane-1-carboxylate synthase 2	Zm00001d002592	GRMZM2G164405	AY359569
ZmERF2	homologue to AtERF1 and 2	Zm00001d002762	GRMZM2G055180	NM_001158578
ZmERF6	homologue to AtERF1 and 2	Zm00001d034920	GRMZM2G381441	NM_001176924
Flavonoid metabolism				
ZmF3H	flavanone 3-hydroxylase	Zm00001d001960	GRMZM2G062396	NM_001112225
Lipase				
ZmPLC	phospholipase C	Zm00001d014903	GRMZM5G889467	NM_001111784
Oxylipin biosynthesis				
ZmLOX3	lipoxygenase 3	Zm00001d033623	GRMZM2G109130	AF329371
ZmLOX4	lipoxygenase 4	Zm00001d033624	GRMZM2G109056	DQ335762
ZmLOX5	lipoxygenase 5	Zm00001d013493	GRMZM2G102760	DQ335763
ZmLOX6	lipoxygenase 6	Zm00001d002000	GRMZM2G040095	DQ335764
ZmLOX9	lipoxygenase 9	Zm00001d027893	GRMZM2G017616	DQ335767
ZmLOX10	lipoxygenase 10	Zm00001d053675	GRMZM2G015419	DQ335768
ZmLOX11	lipoxygenase 11	Zm00001d015852	GRMZM2G009479	DQ335769
ZmAOS2a	allene oxide synthase 2a	Zm00001d028282	GRMZM2G002178	
ZmAOS2b	allene oxide synthase 2b	Zm00001d048021	GRMZM2G067225	AY488135
Transcription factors				
ZmMYC2a	homologue to AtMYC2	Zm00001d007536	GRMZM2G303463	
ZmWRKY14	WRKY transcription factor 14	Zm00001d043569	GRMZM2G040298	EU973705
ZmWRKY46	WRKY transcription factor 46	Zm00001d052357	GRMZM2G063216	EU956406
Repressors of JA signaling				
ZmJAZ1	ZIM domain containing protein	Zm00001d026477	GRMZM2G143402	NM_001156069
ZmJAZ3	ZIM domain containing protein	Zm00001d048268	GRMZM2G036288	NM_001157436
ZmJAZ4	ZIM domain containing protein	Zm00001d027899	GRMZM2G343157	NM_001157673
ZmJAZ5	ZIM domain containing protein	Zm00001d027901	GRMZM2G445634	NM_001156053
ZmJAZ6	ZIM domain containing protein	Zm00001d048263	GRMZM2G036351	NM_001157328
ZmJAZ7	ZIM domain containing protein	Zm00001d014253	GRMZM2G173596	NM_001159100
ZmJAZ8	ZIM domain containing protein	Zm00001d033050	GRMZM2G145412	EU970040
ZmJAZ10	ZIM domain containing protein	Zm00001d020614	GRMZM2G116614	AZM5_3798
ZmJAZ11	ZIM domain containing protein	Zm00001d006860	GRMZM2G101769	AZM5_1941
ZmJAZ12	ZIM domain containing protein	Zm00001d027900	GRMZM5G838098	NM_001154831
ZmJAZ19	ZIM domain containing protein	Zm00001d036494	GRMZM2G080509	AZM5_87469

^†^ Database information: Gramene (http://www.gramene.org/), GRMZM (Maize Genetics and Genomics Database, https://www.maizegdb.org/), Genbank (https://www.ncbi.nlm.nih.gov/genbank/), TIGR (The TIGR Maize Database, http://maize.jcvi.org/).

## References

[1] Awika J.M. (2011). Major cereal grains production and use around the world. Adv. Cereal Sci. Implic. Food Proc. Health Promot..

[2] Food and Agriculture Organization of the United Nations (2013). Food Outlook: Biannual Report of Global Food Markets.

[3] Schnable P.S., Ware D., Fulton R.S., Stein J.C., Wei F., Pasternak S., Liang C., Zhang J., Fulton L., Graves T.A. (2009). The B73 maize genome: Complexity, diversity, and dynamics. Science.

[4] Vielle-Calzada J.P., de la Vega O.M., Hernandez-Guzman G., Ibarra-Laclette E., Alvarez-Mejia C., Vega-Arreguin J.C., Jimenez-Moraila B., Fernandez-Cortes A., Corona-Armenta G., Herrera-Estrella L. (2009). The palomero genome suggests metal effects on domestication. Science.

[5] Andorf C.M., Cannon E.K., Portwood J.L., Gardiner J.M., Harper L.C., Schaeffer M.L., Braun B.L., Campbell D.A., Vinnakota A.G., Sribalusu V.V. (2016). Maizegdb update: New tools, data and interface for the maize model organism database. Nucleic Acids Res..

[6] Kellogg E.A. (2001). Evolutionary history of the grasses. Plant Physiol..

[7] Gowik U., Westhoff P. (2011). The path from C3 to C4 photosynthesis. Plant Physiol..

[8] Vogel J.P., Garvin D.F., Mockler T.C., Schmutz J., Rokhsar D., Bevan M.W., Barry K., Lucas S., Harmon-Smith M., Lail K. (2010). Genome sequencing and analysis of the model grass *brachypodium distachyon*. Nature.

[9] Bennetzen J.L., Schmutz J., Wang H., Percifield R., Hawkins J., Pontaroli A.C., Estep M., Feng L., Vaughn J.N., Grimwood J. (2012). Reference genome sequence of the model plant *Setaria*. Nat. Biotechnol..

[10] Brutnell T.P., Bennetzen J.L., Vogel J.P. (2015). *Brachypodium distachyon* and *Setaria viridis*: Model genetic systems for the grasses. Annu. Rev. Plant Biol..

[11] Estep M.C., McKain M.R., Vela Diaz D., Zhong J., Hodge J.G., Hodkinson T.R., Layton D.J., Malcomber S.T., Pasquet R., Kellogg E.A. (2014). Allopolyploidy, diversification, and the miocene grassland expansion. Proc. Natl. Acad. Sci. USA.

[12] Buczynski M.W., Dumlao D.S., Dennis E.A. (2009). An integrated omics analysis of eicosanoid biology. J. Lipid Res..

[13] Funk C.D. (2001). Prostaglandins and leukotrienes: Advances in eicosanoid biology. Science.

[14] Prost I., Dhondt S., Rothe G., Vicente J., Rodriguez M.J., Kift N., Carbonne F., Griffiths G., Esquerre-Tugaye M.T., Rosahl S. (2005). Evaluation of the antimicrobial activities of plant oxylipins supports their involvement in defense against pathogens. Plant Physiol..

[15] Park S.W., Li W., Viehhauser A., He B., Kim S., Nilsson A.K., Andersson M.X., Kittle J.D., Ambavaram M.M., Luan S. (2013). Cyclophilin 20–3 relays a 12-oxo-phytodienoic acid signal during stress responsive regulation of cellular redox homeostasis. Proc. Natl. Acad. Sci. USA.

[16] v. Euler U.S. (1935). Über die spezifische blutdrucksenkende substanz des menschlichen prostata-und samenblasensekretes. J. Mol. Med..

[17] Goldblatt M.W. (1935). Properties of human seminal plasma. J. Physiol..

[18] Wasternack C. (2015). How jasmonates earned their laurels: Past and present. Plant Growth Regul..

[19] Corey E.J., Weinshenker N.M., Schaaf T.K., Huber W. (1969). Stereo-controlled synthesis of DL-prostaglandins F_2_α and E_2_. J. Am. Chem. Soc.

[20] Fenn J.B., Mann M., Meng C.K., Wong S.F., Whitehouse C.M. (1989). Electrospray ionization for mass spectrometry of large biomolecules. Science.

[21] Pountos I., Georgouli T., Bird H., Giannoudis P.V. (2011). Nonsteroidal anti-inflammatory drugs: Prostaglandins, indications, and side effects. Int. J. Infereron Cytokine Mediator Res..

[22] Vane J.R. (1971). Inhibition of prostaglandin synthesis as a mechanism of action for aspirin-like drugs. Nat.-New Biol..

[23] Meirer K., Steinhilber D., Proschak E. (2014). Inhibitors of the arachidonic acid cascade: Interfering with multiple pathways. Basic Clin. Pharmacol. Toxicol..

[24] Goppelt-Struebe M., Wolter D., Resch K. (1989). Glucocorticoids inhibit prostaglandin synthesis not only at the level of phospholipase A2 but also at the level of cyclo-oxygenase/pge isomerase. Br. J. Pharmacol..

[25] Laine L. (2001). Approaches to nonsteroidal anti-inflammatory drug use in the high-risk patient. Gastroenterology.

[26] U.S. Food and Drug Administration National Drug Code Directory. http://www.fda.gov/Drugs/InformationOnDrugs/ucm142438.htm.

[27] Andreou A., Brodhun F., Feussner I. (2009). Biosynthesis of oxylipins in non-mammals. Prog. Lipid Res..

[28] Hamberg M., Ponce de Leon I., Sanz A., Castresana C. (2002). Fatty acid alpha-dioxygenases. Prostaglandins Other Lipid Mediat..

[29] Mueller M.J. (2004). Archetype signals in plants: The phytoprostanes. Curr. Opin. Plant Biol..

[30] Cuyamendous C., Leung K.S., Durand T., Lee J.C., Oger C., Galano J.M. (2015). Synthesis and discovery of phytofurans: Metabolites of alpha-linolenic acid peroxidation. Chem. Commun. (Camb.).

[31] Schomburg A. Oxylipin Profiling Database. http://www.oxylipins.uni-goettingen.de/path.php?path=Pathway&caption=Oxylipin%20Pathway.

[32] Wasternack C. (2007). Jasmonates: An update on biosynthesis, signal transduction and action in plant stress response, growth and development. Ann. Bot..

[33] Wasternack C., Hause B. (2013). Jasmonates: Biosynthesis, perception, signal transduction and action in plant stress response, growth and development. An update to the 2007 review in annals of botany. Ann. Bot..

[34] Miersch O., Bohlmann H., Wasternack C. (1999). Jasmonates and related compounds from *Fusarium oxysporum*. Phytochemistry.

[35] Miersch O., Bruckner B., Schmidt J., Sembdner G. (1992). Cyclopentane fatty-acids from *Gibberella fujikuroi*. Phytochemistry.

[36] Miersch O., Günther T., Fritsche W., Sembdner G. (1993). Jasmonates from different fungal species. Nat. Prod. Lett..

[37] Tsukada K., Takahashi K., Nabeta K. (2010). Biosynthesis of jasmonic acid in a plant pathogenic fungus, *Lasiodiplodia theobromae*. Phytochemistry.

[38] Melotto M., Underwood W., Koczan J., Nomura K., He S.Y. (2006). Plant stomata function in innate immunity against bacterial invasion. Cell.

[39] Brooks D.M., Bender C.L., Kunkel B.N. (2005). The *Pseudomonas syringae* phytotoxin coronatine promotes virulence by overcoming salicylic acid-dependent defences in *Arabidopsis thaliana*. Mol. Plant Pathol..

[40] Borrego E.J., Kolomiets M.V. (2012). Lipid-mediated signaling between fungi and plants. Biocommunication of Fungi.

[41] Russell W. (1972). Registration of B70 and B73 parental lines of maize (reg. Nos. Pl16 and pl17). Crop Sci..

[42] De La Fuente G.N., Murray S.C., Isakeit T., Park Y.S., Yan Y., Warburton M.L., Kolomiets M.V. (2013). Characterization of genetic diversity and linkage disequilibrium of *ZmLOX4* and *ZmLOX5* loci in maize. PLoS ONE.

[43] Yan Y., Borrego E., Kolomiets M.V. (2013). Jasmonate Biosynthesis, Perception and Function in Plant Development and Stress Responses.

[44] Acosta I.F., Laparra H., Romero S.P., Schmelz E., Hamberg M., Mottinger J.P., Moreno M.A., Dellaporta S.L. (2009). *Tasselseed1* is a lipoxygenase affecting jasmonic acid signaling in sex determination of maize. Science.

[45] Yan Y., Christensen S., Isakeit T., Engelberth J., Meeley R., Hayward A., Emery R.J., Kolomiets M.V. (2012). Disruption of OPR7 and OPR8 reveals the versatile functions of jasmonic acid in maize development and defense. Plant Cell.

[46] Feussner I., Wasternack C. (2002). The lipoxygenase pathway. Annu. Rev. Plant Biol..

[47] Ghanem M.E., Ghars M.A., Frettinger P., Perez-Alfocea F., Lutts S., Wathelet J.P., du Jardin P., Fauconnier M.L. (2012). Organ-dependent oxylipin signature in leaves and roots of salinized tomato plants (*Solanum lycopersicum*). J. Plant Physiol..

[48] Gao X.Q., Stumpe M., Feussner I., Kolomiets M. (2008). A novel plastidial lipoxygenase of maize (*Zea mays*) ZmLOX6 encodes for a fatty acid hydroperoxide lyase and is uniquely regulated by phytohormones and pathogen infection. Planta.

[49] Christensen S.A., Kolomiets M.V. (2011). The lipid language of plant-fungal interactions. Fungal Genet. Biol..

[50] Park Y.S., Kunze S., Ni X., Feussner I., Kolomiets M.V. (2010). Comparative molecular and biochemical characterization of segmentally duplicated 9-lipoxygenase genes ZmLOX4 and ZmLOX5 of maize. Planta.

[51] Christensen S.A., Nemchenko A., Borrego E., Murray I., Sobhy I.S., Bosak L., DeBlasio S., Erb M., Robert C.A., Vaughn K.A. (2013). The maize lipoxygenase, ZmLOX10, mediates green leaf volatile, jasmonate and herbivore-induced plant volatile production for defense against insect attack. Plant J..

[52] Umate P. (2011). Genome-wide analysis of lipoxygenase gene family in *Arabidopsis* and rice. Plant Signal. Behav..

[53] Chauvin A., Caldelari D., Wolfender J.L., Farmer E.E. (2013). Four 13-lipoxygenases contribute to rapid jasmonate synthesis in wounded *Arabidopsis thaliana* leaves: A role for lipoxygenase 6 in responses to long-distance wound signals. New Phytol..

[54] Nemchenko A., Kunze S., Feussner I., Kolomiets M. (2006). Duplicate maize 13-lipoxygenase genes are differentially regulated by circadian rhythm, cold stress, wounding, pathogen infection, and hormonal treatments. J. Exp. Bot..

[55] Wu Q., Luo A., Zadrozny T., Sylvester A., Jackson D. (2013). Fluorescent protein marker lines in maize: Generation and applications. Int. J. Dev. Biol..

[56] Schiller D., Contreras C., Vogt J., Dunemann F., Defilippi B.G., Beaudry R., Schwab W. (2015). A dual positional specific lipoxygenase functions in the generation of flavor compounds during climacteric ripening of apple. Hortic. Res..

[57] Hughes R.K., West S.I., Hornostaj A.R., Lawson D.M., Fairhurst S.A., Sanchez R.O., Hough P., Robinson B.H., Casey R. (2001). Probing a novel potato lipoxygenase with dual positional specificity reveals primary determinants of substrate binding and requirements for a surface hydrophobic loop and has implications for the role of lipoxygenases in tubers. Biochem. J..

[58] Palmieri-Thiers C., Canaan S., Brunini V., Lorenzi V., Tomi F., Desseyn J.L., Garscha U., Oliw E.H., Berti L., Maury J. (2009). A lipoxygenase with dual positional specificity is expressed in olives (*Olea europaea* L.) during ripening. Biochim. Biophys. Acta.

[59] Wang R., Shen W., Liu L., Jiang L., Liu Y., Su N., Wan J. (2008). A novel lipoxygenase gene from developing rice seeds confers dual position specificity and responds to wounding and insect attack. Plant Mol. Biol..

[60] Kim E.S., Choi E., Kim Y., Cho K., Lee A., Shim J., Rakwal R., Agrawal G.K., Han O. (2003). Dual positional specificity and expression of non-traditional lipoxygenase induced by wounding and methyl jasmonate in maize seedlings. Plant Mol. Biol..

[61] Emanuelsson O., Nielsen H., Von Heijne G. (1999). Chlorop, a neural network-based method for predicting chloroplast transit peptides and their cleavage sites. Protein Sci..

[62] Cho K., Han Y., Woo J.C., Baudisch B., Klosgen R.B., Oh S., Han J., Han O. (2011). Cellular localization of dual positional specific maize lipoxygenase-1 in transgenic rice and calcium-mediated membrane association. Plant Sci..

[63] Svitashev S., Young J.K., Schwartz C., Gao H., Falco S.C., Cigan A.M. (2015). Targeted mutagenesis, precise gene editing, and site-specific gene insertion in maize using CAS9 and guide RNA. Plant Physiol..

[64] Itoh A., Schilmiller A.L., McCaig B.C., Howe G.A. (2002). Identification of a jasmonate-regulated allene oxide synthase that metabolizes 9-hydroperoxides of linoleic and linolenic acids. J. Biol. Chem..

[65] Park J.H., Halitschke R., Kim H.B., Baldwin I.T., Feldmann K.A., Feyereisen R. (2002). A knock-out mutation in allene oxide synthase results in male sterility and defective wound signal transduction in *Arabidopsis* due to a block in jasmonic acid biosynthesis. Plant J..

[66] Lee D.S., Nioche P., Hamberg M., Raman C.S. (2008). Structural insights into the evolutionary paths of oxylipin biosynthetic enzymes. Nature.

[67] Christensen S.A., Huffaker A., Kaplan F., Sims J., Ziemann S., Doehlemann G., Ji L., Schmitz R.J., Kolomiets M.V., Alborn H.T. (2015). Maize death acids, 9-lipoxygenase–derived cyclopente (a) nones, display activity as cytotoxic phytoalexins and transcriptional mediators. Proc. Natl. Acad. Sci. USA.

[68] Maucher H., Hause B., Feussner I., Ziegler J., Wasternack C. (2000). Allene oxide synthases of barley (*Hordeum vulgare* cv. Salome): Tissue specific regulation in seedling development. Plant J..

[69] Haga K., Iino M. (2004). Phytochrome-mediated transcriptional up-regulation of allene oxide synthase in rice seedlings. Plant Cell Physiol..

[70] Haga K., Takano M., Neumann R., Iino M. (2005). The rice COLEOPTILE PHOTOTROPISM1 gene encoding an ortholog of *Arabidopsis* NPH3 is required for phototropism of coleoptiles and lateral translocation of auxin. Plant Cell.

[71] Chehab E.W., Raman G., Walley J.W., Perea J.V., Banu G., Theg S., Dehesh K. (2006). Rice hydroperoxide lyases with unique expression patterns generate distinct aldehyde signatures in *Arabidopsis*. Plant Physiol..

[72] Riemann M., Haga K., Shimizu T., Okada K., Ando S., Mochizuki S., Nishizawa Y., Yamanouchi U., Nick P., Yano M. (2013). Identification of rice allene oxide cyclase mutants and the function of jasmonate for defence against *Magnaporthe oryzae*. Plant J..

[73] Yoeun S., Rakwal R., Han O. (2013). Dual positional substrate specificity of rice allene oxide synthase-1: Insight into mechanism of inhibition by type П ligand imidazole. BMB Rep..

[74] Ogorodnikova A.V., Gorina S.S., Mukhtarova L.S., Mukhitova F.K., Toporkova Y.Y., Hamberg M., Grechkin A.N. (2015). Stereospecific biosynthesis of (9*S*,13*S*)-10-oxophytoenoic acid in young maize roots. Biochim. Biophys. Acta Mol. Cell Biol. Lipids.

[75] Bae H.K., Kang H.G., Kim G.J., Eu H.J., Oh S.A., Song J.T., Chung I.K., Eun M.Y., Park S.K. (2010). Transgenic rice plants carrying RNA interference constructs of AOS (allene oxide synthase) genes show severe male sterility. Plant Breed..

[76] Christensen S.A., Nemchenko A., Park Y.S., Borrego E., Huang P.C., Schmelz E.A., Kunze S., Feussner I., Yalpani N., Meeley R. (2014). The novel monocot-specific 9-lipoxygenase ZmLOX12 is required to mount an effective jasmonate-mediated defense against *Fusarium verticillioides* in maize. Mol. Plant Microbe Interact..

[77] Gao X., Starr J., Gobel C., Engelberth J., Feussner I., Tumlinson J., Kolomiets M. (2008). Maize 9-lipoxygenase ZMLOX3 controls development, root-specific expression of defense genes, and resistance to root-knot nematodes. Mol. Plant Microbe Interact..

[78] Engelberth J., Seidl-Adams I., Schultz J.C., Tumlinson J.H. (2007). Insect elicitors and exposure to green leafy volatiles differentially upregulate major octadecanoids and transcripts of 12-oxo phytodienoic acid reductases in *Zea mays*. Mol. Plant Microbe Interact..

[79] Dafoe N.J., Thomas J.D., Shirk P.D., Legaspi M.E., Vaughan M.M., Huffaker A., Teal P.E., Schmelz E.A. (2013). European corn borer (*Ostrinia nubilalis*) induced responses enhance susceptibility in maize. PLoS ONE.

[80] Engelberth J., Contreras C.F., Viswanathan S. (2012). Transcriptional analysis of distant signaling induced by insect elicitors and mechanical wounding in *Zea mays*. PLoS ONE.

[81] Farag M.A., Fokar M., Abd H., Zhang H., Allen R.D., Pare P.W. (2005). (*Z*)-3-hexenol induces defense genes and downstream metabolites in maize. Planta.

[82] Hofmann E., Zerbe P., Schaller F. (2006). The crystal structure of *Arabidopsis thaliana* allene oxide cyclase: Insights into the oxylipin cyclization reaction. Plant Cell.

[83] Wasternack C., Kombrink E. (2010). Jasmonates: Structural requirements for lipid-derived signals active in plant stress responses and development. ACS Chem. Biol..

[84] Stenzel I., Otto M., Delker C., Kirmse N., Schmidt D., Miersch O., Hause B., Wasternack C. (2012). Allene oxide cyclase (AOC) gene family members of *Arabidopsis thaliana*: Tissue- and organ-specific promoter activities and in vivo heteromerization. J. Exp. Bot..

[85] Ziegler J., Wasternack C., Hamberg M. (1999). On the specificity of allene oxide cyclase. Lipids.

[86] Riemann M., Muller A., Korte A., Furuya M., Weiler E.W., Nick P. (2003). Impaired induction of the jasmonate pathway in the rice mutant *hebiba*. Plant Physiol..

[87] Gutjahr C., Gobbato E., Choi J., Riemann M., Johnston M.G., Summers W., Carbonnel S., Mansfield C., Yang S.Y., Nadal M. (2015). Rice perception of symbiotic arbuscular mycorrhizal fungi requires the karrikin receptor complex. Science.

[88] Flematti G.R., Ghisalberti E.L., Dixon K.W., Trengove R.D. (2004). A compound from smoke that promotes seed germination. Science.

[89] Nordstrom K.J., Albani M.C., James G.V., Gutjahr C., Hartwig B., Turck F., Paszkowski U., Coupland G., Schneeberger K. (2013). Mutation identification by direct comparison of whole-genome sequencing data from mutant and wild-type individuals using k-mers. Nat. Biotechnol..

[90] Hazman M., Hause B., Eiche E., Nick P., Riemann M. (2015). Increased tolerance to salt stress in OPDA-deficient rice allene oxide cyclase mutants is linked to an increased ROS-scavenging activity. J. Exp. Bot..

[91] Zhang J., Simmons C., Yalpani N., Crane V., Wilkinson H., Kolomiets M. (2005). Genomic analysis of the 12-oxo-phytodienoic acid reductase gene family of *Zea mays*. Plant Mol. Biol..

[92] Tani T., Sobajima H., Okada K., Chujo T., Arimura S., Tsutsumi N., Nishimura M., Seto H., Nojiri H., Yamane H. (2008). Identification of the OsOPR7 gene encoding 12-oxophytodienoate reductase involved in the biosynthesis of jasmonic acid in rice. Planta.

[93] Schaller F., Biesgen C., Mussig C., Altmann T., Weiler E.W. (2000). 12-oxophytodienoate reductase 3 (OPR3) is the isoenzyme involved in jasmonate biosynthesis. Planta.

[94] Stintzi A., Browse J. (2000). The *Arabidopsis* male-sterile mutant, *opr3*, lacks the 12-oxophytodienoic acid reductase required for jasmonate synthesis. Proc. Natl. Acad. Sci. USA.

[95] Staswick P.E., Tiryaki I. (2004). The oxylipin signal jasmonic acid is activated by an enzyme that conjugates it to isoleucine in *Arabidopsis*. Plant Cell.

[96] Hamberg M., Gardner H.W. (1992). Oxylipin pathway to jasmonates: Biochemistry and biological significance. Biochim. Biophys. Acta.

[97] Miersch O., Kramell R., Parthier B., Wasternack C. (1999). Structure-activity relations of substituted, deleted or stereospecifically altered jasmonic acid in gene expression of barley leaves. Phytochemistry.

[98] Fonseca S., Chini A., Hamberg M., Adie B., Porzel A., Kramell R., Miersch O., Wasternack C., Solano R. (2009). (+)-7-iso-jasmonoyl-l-isoleucine is the endogenous bioactive jasmonate. Nat. Chem. Biol..

[99] Staswick P.E., Tiryaki I., Rowe M.L. (2002). Jasmonate response locus JAR1 and several related *Arabidopsis* genes encode enzymes of the firefly luciferase superfamily that show activity on jasmonic, salicylic, and indole-3-acetic acids in an assay for adenylation. Plant Cell.

[100] Wakuta S., Suzuki E., Saburi W., Matsuura H., Nabeta K., Imai R., Matsui H. (2011). OsJAR1 and OsJAR2 are jasmonyl-l-isoleucine synthases involved in wound- and pathogen-induced jasmonic acid signalling. Biochem. Biophys. Res. Commun..

[101] Riemann M., Riemann M., Takano M. (2008). Rice JASMONATE RESISTANT 1 is involved in phytochrome and jasmonate signalling. Plant Cell Environ..

[102] Xiao Y., Chen Y., Charnikhova T., Mulder P.P., Heijmans J., Hoogenboom A., Agalou A., Michel C., Morel J.B., Dreni L. (2014). OsJAR1 is required for JA-regulated floret opening and anther dehiscence in rice. Plant Mol. Biol..

[103] Shimizu T., Miyamoto K., Miyamoto K., Minami E., Nishizawa Y., Iino M., Nojiri H., Yamane H., Okada K. (2013). OsJAR1 contributes mainly to biosynthesis of the stress-induced jasmonoyl-isoleucine involved in defense responses in rice. Biosci. Biotechnol. Biochem..

[104] Hamberg M. (2000). New cyclopentenone fatty acids formed from linoleic and linolenic acids in potato. Lipids.

[105] Christensen S.A., Huffaker A., Hunter C.T., Alborn H.T., Schmelz E.A. (2015). A maize death acid, 10-oxo-11-phytoenoic acid, is the predominant cyclopentenone signal present during multiple stress and developmental conditions. Plant Signal. Behav..

[106] Matsui K. (2006). Green leaf volatiles: Hydroperoxide lyase pathway of oxylipin metabolism. Curr. Opin. Plant Biol..

[107] Matsui K., Sugimoto K., Mano J., Ozawa R., Takabayashi J. (2012). Differential metabolisms of green leaf volatiles in injured and intact parts of a wounded leaf meet distinct ecophysiological requirements. PLoS ONE.

[108] Howe G.A., Jander G. (2008). Plant immunity to insect herbivores. Annu. Rev. Plant Biol..

[109] Schuman M.C., Baldwin I.T. (2016). The layers of plant responses to insect herbivores. Annu. Rev. Entomol..

[110] War A.R., Paulraj M.G., Ahmad T., Buhroo A.A., Hussain B., Ignacimuthu S., Sharma H.C. (2012). Mechanisms of plant defense against insect herbivores. Plant Signal. Behav..

[111] Turlings T.C.J., Alborn H.T., Loughrin J.H., Tumlinson J.H. (2000). Volicitin, an elicitor of maize volatiles in oral secretion of *Spodoptera exigua*: Isolation and bioactivity. J. Chem. Ecol..

[112] Lait C.G., Alborn H.T., Teal P.E., Tumlinson J.H. (2003). Rapid biosynthesis of *N*-linolenoyl-l-glutamine, an elicitor of plant volatiles, by membrane-associated enzyme(s) in *Manduca sexta*. Proc. Natl. Acad. Sci. USA.

[113] Schmelz E.A., Engelberth J., Alborn H.T., Tumlinson J.H., Teal P.E. (2009). Phytohormone-based activity mapping of insect herbivore-produced elicitors. Proc. Natl. Acad. Sci. USA.

[114] Maes L., Goossens A. (2010). Hormone-mediated promotion of trichome initiation in plants is conserved but utilizes species and trichome-specific regulatory mechanisms. Plant Signal. Behav..

[115] Velez-Bermudez I.C., Salazar-Henao J.E., Fornale S., Lopez-Vidriero I., Franco-Zorrilla J.M., Grotewold E., Gray J., Solano R., Schmidt W., Pages M. (2015). A MYB/ZML complex regulates wound-induced lignin genes in maize. Plant Cell.

[116] Niemeyer H.M. (2009). Hydroxamic acids derived from 2-hydroxy-2 h-1, 4-benzoxazin-3 (4 h)-one: Key defense chemicals of cereals. Agric. Food Chem..

[117] Frey M., Spiteller D., Boland W., Gierl A. (2004). Transcriptional activation of IGL, the gene for indole formation in *Zea mays*: A structure-activity study with elicitor-active n-acyl glutamines from insects. Phytochemistry.

[118] Dafoe N.J., Huffaker A., Vaughan M.M., Duehl A.J., Teal P.E., Schmelz E.A. (2011). Rapidly induced chemical defenses in maize stems and their effects on short-term growth of *Ostrinia nubilalis*. J. Chem. Ecol..

[119] Yang Y.-L., Chang F.-R., Wu C.-C., Wang W.-Y., Wu Y.-C. (2002). New ent-kaurane diterpenoids with anti-platelet aggregation activity from *Annona quamosa*. J. Nat. Prod..

[120] Schmelz E.A., Kaplan F., Huffaker A., Dafoe N.J., Vaughan M.M., Ni X., Rocca J.R., Alborn H.T., Teal P.E. (2011). Identity, regulation, and activity of inducible diterpenoid phytoalexins in maize. Proc. Natl. Acad. Sci. USA.

[121] Mohan S., Ma P.W., Pechan T., Bassford E.R., Williams W.P., Luthe D.S. (2006). Degradation of the *S. frugiperda* peritrophic matrix by an inducible maize cysteine protease. J. Insect Physiol..

[122] Lopez L., Camas A., Shivaji R., Ankala A., Williams P., Luthe D. (2007). MIR1-CP, a novel defense cysteine protease accumulates in maize vascular tissues in response to herbivory. Planta.

[123] Pechan T., Ye L., Chang Y., Mitra A., Lin L., Davis F.M., Williams W.P., Luthe D.S. (2000). A unique 33-kd cysteine proteinase accumulates in response to larval feeding in maize genotypes resistant to fall armyworm and other Lepidoptera. Plant Cell.

[124] Ankala A., Luthe D.S., Williams W.P., Wilkinson J.R. (2009). Integration of ethylene and jasmonic acid signaling pathways in the expression of maize defense protein MIR1-CP. Mol. Plant Microbe Interact..

[125] Louis J., Basu S., Varsani S., Castano-Duque L., Jiang V., Williams W.P., Felton G.W., Luthe D.S. (2015). Ethylene contributes to MAIZE INSECT RESISTANCE1-mediated maize defense against the phloem sap-sucking corn leaf aphid. Plant Physiol..

[126] Tamayo M.C., Rufat M., Bravo J.M., San Segundo B. (2000). Accumulation of a maize proteinase inhibitor in response to wounding and insect feeding, and characterization of its activity toward digestive proteinases of *Spodoptera littoralis* larvae. Planta.

[127] Zhu-Salzman K., Zeng R. (2015). Insect response to plant defensive protease inhibitors. Annu. Rev. Entomol..

[128] Farmer E.E., Johnson R.R., Ryan C.A. (1992). Regulation of expression of proteinase inhibitor genes by methyl jasmonate and jasmonic acid. Plant Physiol..

[129] Farmer E.E., Ryan C.A. (1992). Octadecanoid precursors of jasmonic acid activate the synthesis of wound-inducible proteinase inhibitors. Plant Cell.

[130] Yan J., Lipka A.E., Schmelz E.A., Buckler E.S., Jander G. (2014). Accumulation of 5-hydroxynorvaline in maize (*Zea mays*) leaves is induced by insect feeding and abiotic stress. J. Exp. Bot..

[131] Dabrowska P., Freitak D., Vogel H., Heckel D.G., Boland W. (2009). The phytohormone precursor OPDA is isomerized in the insect gut by a single, specific glutathione transferase. Proc. Natl. Acad. Sci. USA.

[132] Shabab M., Khan S.A., Vogel H., Heckel D.G., Boland W. (2014). Opda isomerase GST16 is involved in phytohormone detoxification and insect development. FEBS J..

[133] Heil M. (2008). Indirect defence via tritrophic interactions. New Phytol..

[134] Schmelz E.A., Alborn H.T., Banchio E., Tumlinson J.H. (2003). Quantitative relationships between induced jasmonic acid levels and volatile emission in *Zea mays* during *Spodoptera exigua* herbivory. Planta.

[135] Kollner T.G., Lenk C., Schnee C., Kopke S., Lindemann P., Gershenzon J., Degenhardt J. (2013). Localization of sesquiterpene formation and emission in maize leaves after herbivore damage. BMC Plant Biol..

[136] Schmelz E.A., Alborn H.T., Tumlinson J.H. (2003). Synergistic interactions between volicitin, jasmonic acid and ethylene mediate insect-induced volatile emission in *Zea mays*. Physiol. Plant..

[137] Ozawa R., Shiojiri K., Sabelis M.W., Arimura G., Nishioka T., Takabayashi J. (2004). Corn plants treated with jasmonic acid attract more specialist parasitoids, thereby increasing parasitization of the common armyworm. J. Chem. Ecol..

[138] Ozawa R., Shiojiri K., Sabelis M.W., Takabayashi J. (2008). Maize plants sprayed with either jasmonic acid or its precursor, methyl linolenate, attract armyworm parasitoids, but the composition of attractants differs. Entomol. Exp. Appl..

[139] Von Mérey G.E., Veyrat N., de Lange E., Degen T., Mahuku G., Valdez R.L., Turlings T.C., D’Alessandro M. (2012). Minor effects of two elicitors of insect and pathogen resistance on volatile emissions and parasitism of *Spodoptera frugiperda* in mexican maize fields. Biol. Control..

[140] Kim J., Felton G.W. (2013). Priming of antiherbivore defensive responses in plants. Insect Sci..

[141] Ali M., Sugimoto K., Ramadan A., Arimura G. (2013). Memory of plant communications for priming anti-herbivore responses. Sci. Rep..

[142] Jaskiewicz M., Conrath U., Peterhansel C. (2011). Chromatin modification acts as a memory for systemic acquired resistance in the plant stress response. EMBO Rep..

[143] Engelberth J., Alborn H.T., Schmelz E.A., Tumlinson J.H. (2004). Airborne signals prime plants against insect herbivore attack. Proc. Natl. Acad. Sci. USA.

[144] Ton J., D’Alessandro M., Jourdie V., Jakab G., Karlen D., Held M., Mauch-Mani B., Turlings T.C. (2007). Priming by airborne signals boosts direct and indirect resistance in maize. Plant J..

[145] Erb M., Veyrat N., Robert C.A., Xu H., Frey M., Ton J., Turlings T.C. (2015). Indole is an essential herbivore-induced volatile priming signal in maize. Nat. Commun..

[146] Oluwafemi S., Dewhirst S.Y., Veyrat N., Powers S., Bruce T.J., Caulfield J.C., Pickett J.A., Birkett M.A. (2013). Priming of production in maize of volatile organic defence compounds by the natural plant activator cis-jasmone. PLoS ONE.

[147] Glazebrook J. (2005). Contrasting mechanisms of defense against biotrophic and necrotrophic pathogens. Annu. Rev. Phytopathol..

[148] Vaughan M.M., Huffaker A., Schmelz E.A., Dafoe N.J., Christensen S., Sims J., Martins V.F., Swerbilow J., Romero M., Alborn H.T. (2014). Effects of elevated [CO_2_] on maize defence against mycotoxigenic *Fusarium verticillioides*. Plant Cell Environ..

[149] Tang J.D., Perkins A., Williams W.P., Warburton M.L. (2015). Using genome-wide associations to identify metabolic pathways involved in maize aflatoxin accumulation resistance. BMC Genom..

[150] Gao X., Brodhagen M., Isakeit T., Brown S.H., Gobel C., Betran J., Feussner I., Keller N.P., Kolomiets M.V. (2009). Inactivation of the lipoxygenase ZmLOX3 increases susceptibility of maize to *Aspergillus* spp. Mol. Plant Microbe Interact..

[151] Huffaker A., Dafoe N.J., Schmelz E.A. (2011). Zmpep1, an ortholog of *Arabidopsis* elicitor peptide 1, regulates maize innate immunity and enhances disease resistance. Plant Physiol..

[152] Stenzel I., Hause B., Maucher H., Pitzschke A., Miersch O., Ziegler J., Ryan C.A., Wasternack C. (2003). Allene oxide cyclase dependence of the wound response and vascular bundle-specific generation of jasmonates in tomato–amplification in wound signalling. Plant J..

[153] Pieterse C.M., Leon-Reyes A., Van der Ent S., Van Wees S.C. (2009). Networking by small-molecule hormones in plant immunity. Nat. Chem. Biol..

[154] Constantino N.N., Mastouri F., Damarwinasis R., Borrego E.J., Moran-Diez M.E., Kenerley C.M., Gao X., Kolomiets M.V. (2013). Root-expressed maize LIPOXYGENASE 3 negatively regulates induced systemic resistance to *Colletotrichum graminicola* in shoots. Front. Plant Sci..

[155] Planchamp C., Glauser G., Mauch-Mani B. (2014). Root inoculation with *Pseudomonas putida* KT2440 induces transcriptional and metabolic changes and systemic resistance in maize plants. Front. Plant Sci..

[156] Neal A.L., Ton J. (2013). Systemic defense priming by *Pseudomonas putida* KT2440 in maize depends on benzoxazinoid exudation from the roots. Plant Signal. Behav..

[157] Yamane H., Sugawara J., Suzuki Y., Shimamura E., Takahashi N. (1980). Syntheses of jasmonic acid related-compounds and their structure-activity-relationships on the growth of rice seedings. Agric. Biol. Chem..

[158] Yan Y., Huang P.-C., Borrego E., Kolomiets M. (2014). New perspectives into jasmonate roles in maize. Plant Signal. Behav..

[159] Calderon-Urrea A., Dellaporta S.L. (1999). Cell death and cell protection genes determine the fate of pistils in maize. Development.

[160] DeLong A., Calderon-Urrea A., Dellaporta S.L. (1993). Sex determination gene TASSELSEED 2 of maize encodes a short-chain alcohol dehydrogenase required for stage-specific floral organ abortion. Cell.

[161] Wu X., Knapp S., Stamp A., Stammers D.K., Jornvall H., Dellaporta S.L., Oppermann U. (2007). Biochemical characterization of TASSELSEED 2, an essential plant short-chain dehydrogenase/reductase with broad spectrum activities. FEBS J..

[162] Hartwig T., Chuck G.S., Fujioka S., Klempien A., Weizbauer R., Potluri D.P., Choe S., Johal G.S., Schulz B. (2011). Brassinosteroid control of sex determination in maize. Proc. Natl. Acad. Sci. USA.

